# Enhancing Clinical Depression Treatment Outcomes: A Comprehensive Approach Using Measurement-Based Care, Research Benchmarks, and Systematic Quality Improvements

**DOI:** 10.1007/s10488-026-01499-6

**Published:** 2026-03-31

**Authors:** Fabian Lenhard, Lisa Wahlström Amnéus, Ida Viklund, Erik Andersson, Lars-Göran Öst

**Affiliations:** 1 Centre for Psychiatry Research, Department of Clinical Neuroscience, Karolinska Institutet, & Stockholm Health Care Services, Region Stockholm, Stockholm, Sweden; 2 WeMind, Stockholm, Sweden; 3 Division of Psychology, Department of Clinical Neuroscience, Karolinska Institutet, Stockholm, Sweden; 4 Department of Psychology, Stockholm University, Stockholm, Sweden

**Keywords:** Depression, Measurement-based care, Patient-reported outcome measures, Quality improvement, Benchmarking

## Abstract

Depression is a leading cause of global disease burden. Although psychotherapy and pharmacotherapy effectively reduce depressive symptoms, access to high-quality care remains limited. This study examined whether a Continuous Quality Improvement (CQI) process incorporating measurement-based care and benchmarking could enhance depression treatment outcomes in specialized mental healthcare. Data were drawn from two outpatient mental health clinics in Stockholm, Sweden. First, patient-reported outcome measures (PROMs) were used to compare local clinical results with a meta-analytic benchmark. Next, a CQI framework was implemented to improve outcomes. The sample included 415 patients receiving treatment for depression. Compared to the benchmark, the clinical sample had higher pre-treatment depression severity, attended more appointments, and had lower data attrition. Initial year-to-year analyses showed that treatment effects were significantly lower than the benchmark (Hedge’s g = 0.87–1.01 vs. g = 1.51). After implementing the CQI process, treatment effects improved (g = 1.25–1.27) and were no longer inferior to the benchmark. These findings suggest that integrating measurement-based care, benchmarking, and CQI can improve depression treatment outcomes in specialized settings and may help close the gap between real-world care and evidence-based standards.

**Supplementary Information:**

The online version contains supplementary material available at 10.1007/s10488-026-01499-6.

## Background

Depression is among the leading causes of disease burden worldwide (Vos et al., 2020). The World Health Organization (WHO) has ranked clinical depression as the largest contributor to global disability, affecting over 300 million people and causing 7.5% of all years lived with disability (WHO, 2017). Moreover, depression is one of the primary factors contributing to suicide deaths (Vigo et al., 2016). The impact of clinical depression on everyday life is profound and impairs an individual’s ability to function in various domains, including work, social interactions, and personal relationships (Kessler, 2012). It is associated with low educational attainment, unstable employment as well as a higher risk for both somatic and psychiatric comorbidities (Kessler, 2012).

Evidence-based treatment options for clinical depression include Cognitive Behavior Therapy (CBT) and pharmacotherapy, either as mono-therapies or in combination (American Psychological Association, 2019; National Board of Health and Welfare, 2019; NICE, 2022). A recent network meta-analysis reported a moderate standardized mean difference (MSD = 0.57 (95% confidence interval [0.08–1.07]) for CBT in reducing depressive symptoms (Ciharova et al., 2021), demonstrating its efficacy compared to control conditions. However, previous analyses of efficacy studies indicated that the effects of CBT for depression may be somewhat lower when implemented in routine clinical practice (Hans & Hiller, 2013).

Pharmacotherapy primarily involves Selective Serotonin Reuptake Inhibitors (SSRIs). Cipriani et al. (2018) found response rate odds ratios between OR = 1.37 to 2.13 for SSRIs compared to pill placebo, corresponding to an standardized effect size of Cohen’s d = 0.17 to 0.46 (Sánchez-Meca et al., 2003). Combining CBT with pharmacotherapy can enhance treatment outcomes, particularly in moderate to severe depression, with studies suggesting synergistic effects (Cuijpers et al., 2009).

While evidence-based treatment strategies have been proven effective for clinical depression, only a minority of individuals with the condition actually receive treatment, and the quality of care provided to the majority of those undergoing treatment remains unacceptably low (Thornicroft et al., 2017). In a WHO-led study of 21 countries, only one in five people in high-income and one in 27 in low-/lower-middle-income countries received minimally adequate treatment (Thornicroft et al., 2017). Importantly, quality of care for mental health conditions has not increased to the same extent as that for physical conditions (Kilbourne et al., 2018). Thus, improvements in accessibility as well as the quality of care are crucial.

The clinical application of routinely administered outcome measurements (measurement-based care, MBC) in combination with benchmarking and continuous quality improvements (CQI) methodologies has been advocated as tools for improving clinical outcomes in mental healthcare (Bramesfeld et al., 2016; Endalamaw et al., 2024; Kilbourne et al., 2018). Core principles of MBC are the collection of clinical outcome measures (such as the PHQ-9 for depressive symptoms (Kroenke et al., 2001), the systematic evaluation of patient-reported symptoms before or during an encounter, and a collaborative involvement of the patient in the follow-up and further planning of treatment, based on these measurements (Zhu et al., 2021).

The essence of benchmarking is to compare group- or clinic-level outcomes with a chosen gold standard, to monitor the current status of relevant indicators of quality and identify areas for improvement (Bayney, 2005). Previous studies have demonstrated that benchmarking methodology can be used to compare outcomes between mental health services (Delgadillo et al., 2014), different treatment modalities of psychological treatments (Wickberg et al., 2022) as well as comparisons on a national level (Bramesfeld et al., 2016). A recent meta-analysis from the somatic conditions field demonstrated that the use of benchmarking was significantly associated with improvements in process and treatment outcomes (Willmington et al., 2022). However, results that demonstrate the value of benchmarking for improvements in mental healthcare are scarce.

CQI involves a set of different methodologies with common features such as the identification of areas in need of improvement, the development and execution of a strategy aimed towards improvement and a follow-up of implemented actions or procedures. Previous research has demonstrated that systematic CQI approaches can improve clinical outcomes and overall quality of care in the treatment of depression in primary care (Wells et al., 2000). However, results of treatment of depression from specialized psychiatric settings, studying the combination of MBC, benchmarking and CQI, are currently not available.

The aim of the current study was to evaluate a CQI process, guided by MBC and benchmarking data, targeting the improvement of depression treatment outcomes within a specialized mental healthcare environment.

## Method

This study was approved by Swedish Ethical Review Authority and was exempted from participant consent as all data was collected according to standard clinical routines. The study is reported according to SQUIRE 2.0 standards (Davidoff et al., 2009).

### Setting

The data used in this study originate from two outpatient mental health clinics, located in the inner-city area of Stockholm, Sweden. The clinics are operated by WeMind, a mental healthcare provider commissioned by Region Stockholm. Both clinics are specialized in the psychiatric treatment of depression as well as anxiety disorders, OCD and related disorders, and PTSD. Treatments were provided according to standard procedures within the two clinics. These clinical procedures were implemented in line with national clinical guidelines (National Board of Health and Welfare, 2019; Region Stockholm, 2024), supporting clinicians as to which evidence-based pharmacological and psychological treatments should be offered under different clinical scenarios. Typically, patients received cognitive behavior therapy (CBT), pharmacotherapy or a combination of both. Clinicians were experienced licensed psychologists and licensed psychiatrists.

### Procedures

Data collection procedures followed the regular clinical assessment routines established at the participating clinics. No additional elements were added for research purposes. Patients were adult individuals referred from primary (general practitioner) and secondary (specialized) healthcare services to either clinic for initial diagnostic assessment. Prior to the first visit, patients were assessed with standardized, self-rating measurement batteries regarding overall symptoms and functional impairment. Patients were then invited to a first appointment at the clinic. During this intake visit, a semi-structured psychiatric assessment was conducted to establish the primary diagnosis and comorbidities, using the Mini International Neuropsychiatric Interview (MINI) (Sheehan et al., 1998). Patients were then offered treatment alternatives dependent on their primary mental health condition and comorbidity profile. In conjunction with the start of treatment, a standardized battery of PROMs was administered. PROMs were administered once per week during treatment but could be individually adjusted by the treating clinician. Each measurement battery included a primary diagnosis-specific PROM (see “Measures” section). Patients completed the measurements online via a personal, secure login, using two-way authentication. The post-treatment PROM assessment was defined as the measurement closest to the end of treatment, however with the requirement of at least 6 weeks separation from the pre-treatment measurement time point.

### A Comprehensive Model for the Combination of MBC, Benchmarking and CQI

**Fig. 1:**
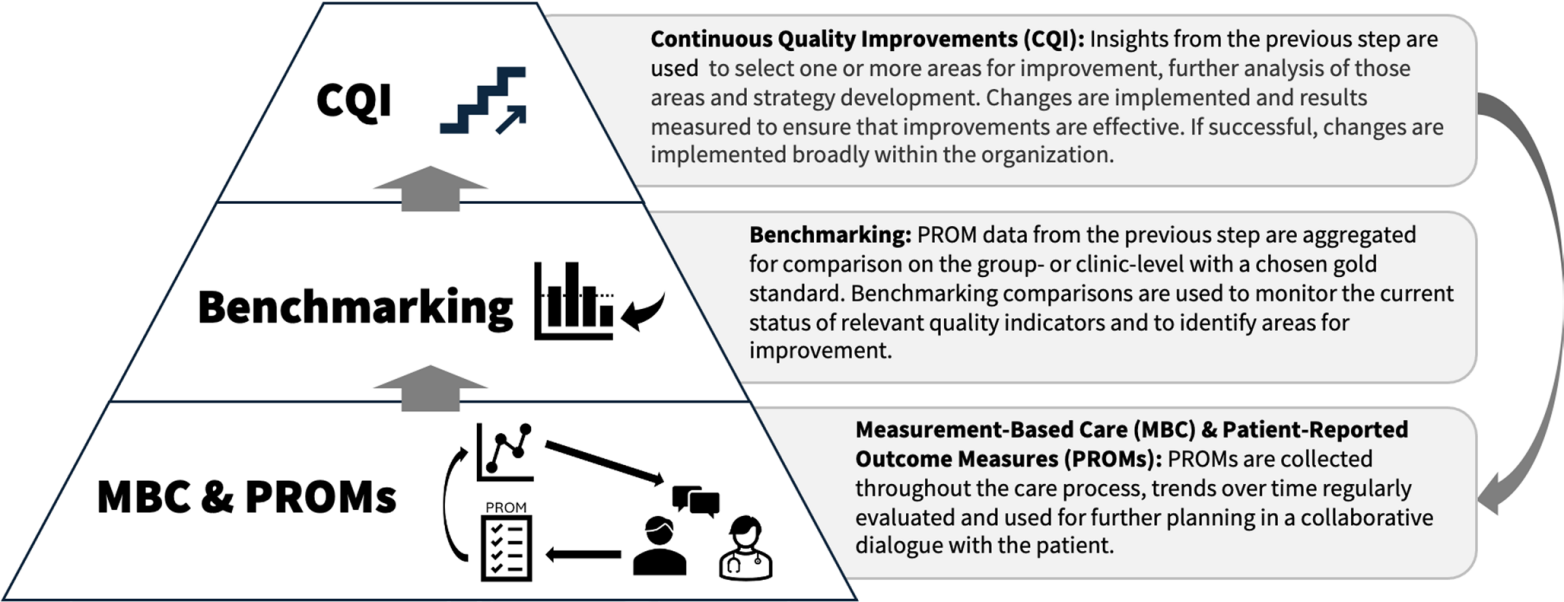
A comprehensive model for the combination of measurement-based care, benchmarking, and continuous quality improvements

The CQI process was guided by a comprehensive model, combining principles of MBC, benchmarking and CQI (Endalamaw et al., 2024). Figure 1 summarizes the model.

### MBC

Since 2008, principles of MBC (Lewis et al., 2019) had been implemented, including the continuous collection of diagnosis-specific patient-reported outcome measures (PROMs). For follow-up of the treatment of patients with depression the PHQ-9 (Kroenke et al., 2001) was the primary measure. Therefore, relevant outcome data were available with sufficient coverage on a year-to-year basis.

### Benchmarking

A benchmarking audit was conducted during the years 2019 through 2021. Data from all available pre- and post-treatment measurements of PHQ-9 were compiled via an electronic assessment tool and visually presented to the clinic managers and staff. The meta-analysis of Hans and Hiller (2013) was used as a gold standard comparison of the clinical outcomes. However, as there was more comprehensive and updated data available in a more recent publication, we then used the meta-analysis by Öst and co-workers in our statistical analyses (Öst et al. 2023a). This meta-analysis included both RCTs and pre-post trials in adult populations of depressed patients, which enabled effect size calculations of within-group change of disorder-specific depressive symptoms. This meta-analysis used both interviewer-based ratings and self-rating measures. However, since the clinical data included in the current study only used patient-reported measures, we included only studies from the meta-analysis that provided data on a reliable and valid patient report measure of depressive symptoms. For a detailed description, see the original publication (Öst et al. 2023a).

### Quality Control and Improvement Procedure

During the benchmarking audit in 2019 to 2021, the clinic managers identified the treatment outcomes for depression as an area for improvement, as the outcomes were below the chosen benchmark (Hans & Hiller, 2013). As a result of this decision, a quality improvement cycle was initiated.

The CQI process that was used in the current case was the FOCUS-PDCA cycle (Endalamaw et al., 2024), consisting of the following steps: Find a process to improve (F), organize a knowledgeable team (O), clarify current knowledge of the process (C), understand sources of process variations (U), and select improvements (S), plan an approach (P), do the activity (D), check the results (C) and act on the results (A).

As a first step of the FOCUS-PDA cycle, the quality assurance manager (coauthor FL) together with the clinic managers (coauthors LWA, IW) identified that depression treatment outcomes were not in line with the research benchmark (Hans & Hiller, 2013). A clinical team of three clinical psychologists and two psychiatrists was organized (O) to clarify the knowledge regarding current diagnostic and treatment routines for patients with depression (C). To gain a better understanding of process variations and areas for improvement (U), a systematic review of medical records of completed depression treatments was carried out by the clinical team. This review resulted in two insights:

1. Instead of one patient population of individuals with clinical depression, when the onset and course of symptoms were considered more closely, a categorization into three distinct sub-groups appeared more appropriate: (A) Patients with a primary diagnosis of major depression, (B) Patients with depression as a comorbid condition to an anxiety disorder, (C) Patients with depression as a comorbid condition to a personality disorder. The three diagnostic sub-groups were clinically defined according to debut (onset characteristics), course (temporal pattern and stability) and previous treatments (response patterns and help-seeking history). Table 1 gives and overview of the clinical categorization on these dimensions.
2. If diagnostic routines were modified according to this subcategorization, the improved clinical assessments should lead to more appropriate allocation of patients to effective treatment options, and thus promote improved treatment outcomes.

**Table 1:** Clinical categorization of the three diagnostic sub-groups on the dimensions of debut, course and previous treatment

	(A) Primary major depression	(B) Depression as a comorbid condition to an anxiety disorder	(C) Depression as a comorbid condition to a personality disorder
Debut	- clear change from premorbid functioning - no longstanding maladaptive personality pattern	- depression develops secondary to chronic anxiety burden - frequently early onset anxiety (childhood / adolescence)	- early and pervasive interpersonal dysfunction - mood instability or chronic dysphoria from adolescence
Course	- often episodic with subclinical or no symptoms between episodes or return to baseline functioning	- depressive episodes recur during anxiety exacerbations - partial remission of mood, but persistent anxiety	- chronic dysphoria rather than distinct episodes - interpersonal crises trigger mood collapses
Previous treatment	- good response to antidepressants and / or psychotherapy	- residual anxiety symptoms persist if not explicitly addressed in treatment	- recurrent treatment failures if focused on depressive symptoms only - therapy alliance instability

The team then selected the above two aspects as relevant improvements (S). The results from the systematic analysis of the medical records were presented to and jointly discussed with the clinical staff at the two clinics. A schedule was planned for the roll out of updated routines for diagnostic and clinical assessments as well as treatment allocation (P). The new routines were implemented in the second half of 2021 (D) and followed-up (C) in 2022 and 2023, see Results section below. Due to the positive results of this quality improvement cycle, the results were then implemented as standard routines at the clinics as well as shared with other clinics in the organization (A).

### Assessment

#### Measures

Participants were assessed before and after treatment. For the clinical outcome measurements of clinical depression the Patient Health Questionnaire-9 (PHQ-9, Kroenke et al., 2001) was used.

#### Background Variables

*Gender*. Since the lifetime prevalence for depressive disorders is higher for women than men we record this variable as percent females (Hasin et al., 2018). *Mean age*. There is no indication that the age of the participants is a significant predictor of outcome among adults, but it is presented as a basic background variable. *Number of treatment sessions*. It is important when comparing studies in routine clinical care and research settings that the number of therapy sessions does not deviate substantially from what the developers of CBT methods designed, i.e., 12–16 sessions. Thus, it is important to record the number of therapy sessions. *Pre-treatment severity*. It is well-known that the pre-treatment score on the outcome measure is a significant predictor of the post-treatment score (Öst et al. 2023a, 2023b). We recorded the sample’s mean score as percent of the maximum score possible on the instrument in question.

### Statistical Analyses

Year-by-year treatment outcomes on the PHQ-9 were compared with the most recent meta-analytic benchmark (Öst et al. 2023a). The meta-analytic benchmark study included different self-report measures. Since all the measures were reliable and valid as patient-reported measures of depression, we pooled their pre- and post-treatment results to derive a mean effect size for the respective disorder. Within-group effect size (ES) was calculated as (Mpre − Mpost)/SDpre according to recommendation by Lakens (2013), as there is good reason to assume that the interventions influence not only the means but also the standard deviations. The mean ES was computed by weighting each ES by the inverse of its variance. When a study presented intent-to-treat data these were used, if not completer data were used. The software *Comprehensive Meta-Analysis v.4* (Borenstein et al., 2022) was used for all analyses, and to correct for small sample sizes, Hedges’ g was calculated.

We compared the means on background variables and mean ES for the outcome variables in the following way. If the clinic’s mean was within the 95% confidence interval (CI) of the meta-analysis we considered it not a significant difference, and if the mean was below or above the CI we considered it a significant difference. Whether the clinical mean was better or worse depended on the individual background variable, whereas for ES a mean below the CI means a significantly worse, and above the CI a significantly better outcome. Regarding the year-by-year analysis of treatment outcomes, we considered a significant difference if the clinical and benchmark confidence intervals did not overlap and non-significant if they did overlap.

## Results

### Background Data

The total sample consisted of *N* = 415 patients, of which 353 (85,3%) provided valid pre- and post-treatment measurements. A comparison between the clinical and meta-analytic benchmark samples on background variables is presented in Table 2. There was no difference regarding percent female patients and mean age of the participants. However, the clinical sample had a higher pre-treatment depression severity score, received more therapy sessions, and had a lower attrition rate than the meta-analytic benchmark.

**Table 2:** Background variables for the clinical and benchmark samples

Sample	k	*N*	% females	Mean age	% Pre Tx severity	Mean number of sessions	% data attrition
Clinical	1	415	63.4	38.2 (SD = 13.2)	58.7 H	16.1 (SD = 13.2) H	14.7 L
Benchmark	32	3525	66.4	39.1	48.3	12.7	23.7
(95% CI)			(62.8–70.0)	(36.5–41.7)	(45.1–51.4)	(10.9–14.4)	(17.9–29.5)

*k* = number of samples or studies. N = number of participants. CI = confidence interval. Tx = treatment. L = lower than benchmark, H = higher than benchmark

### Year-by-Year Analysis

**Fig. 2:**
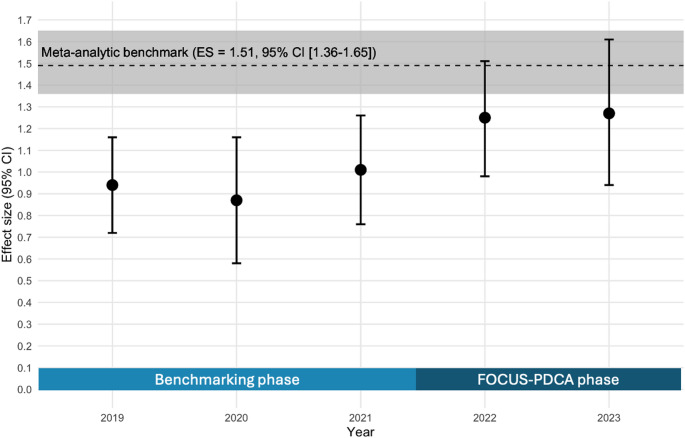
Annual effect sizes (ES) and 95% confidence intervals (CI) for depression treatments compared to the meta-analytic benchmark (Öst et al., 2023a)

Figure 2 shows the effects of depression treatments divided by year, in comparison with the mean pre-post ES of 1.51 for the effectiveness studies (see as well Supplementary materials Table S1). Clinical treatment effects were significantly lower than that of MA effectiveness studies in 2019 to 2021. For 2022 and 2023 the treatment effect was non-inferior to the MA studies.

## Discussion

The aim of this study was to evaluate a quality improvement cycle targeted on depression treatment outcomes. The quality improvement process was based on MBC data collected in specialized outpatient mental healthcare and a meta-analytic research benchmark. We found that treatment outcomes improved as findings from the benchmarking and CQI phases were implemented in clinical practice. As a result, treatment outcomes were on par with the meta-analytic benchmark in 2022 and 2023.

While the best available benchmark was used for the benchmarking audit in the initial phase of the project (Hans & Hiller, 2013), we then used a more recent and more comprehensive meta-analysis for the analyses (Öst et al., 2023a). In addition, the meta-analysis of Öst et al. (2023a) provided a more conservative benchmark, with an effect size of d = 1.51, compared to that of Hans and Hiller (2013), g = 1.26.

We think several insights can be drawn from this study. Firstly, as MBC was implemented several years before this study, good quality data on PROMs were readily available. This availability of relevant outcome data, in this case PHQ-9 measurements from pre- and post-treatment, made it possible for the team of clinic managers, quality assurance staff and researchers to conduct a benchmark with meta-analytic results. Thus, the routinely implemented collection of PROMs was a key prerequisite for a systematic comparison with a research benchmark, which in turn is a necessity to identify areas for improvement. However, previous studies indicated that the use of MBC and routinely collected measures is widely underutilized, with only 13.9% of clinicians using such measures at least monthly and the majority, 61.5%, never uses them (Jensen-Doss et al., 2018). In our study, 85% of patients contributed with valid pre- and post-treatment measurements, indicating a high level of implementation of MBC at the participating clinics and sufficient data quality to support confidence in our analyses. Efforts to better understand barriers for implementation and to facilitate the use of PROMs are therefore an important first step.

Secondly, the application of a FOCUS-PDCA cycle resulted in modifications of diagnostic routines, which preceded significant improvements in clinical outcomes. Whereas the study design does not allow causal inference, the improvements in outcome were in close temporal association with improvements made in the clinical routines. A possible next step would be to replicate the findings from our study with an experimental control group design, e.g., by randomizing clinics to a CQI condition and compare treatment outcomes to clinics without such an intervention.

Thirdly, the improvements in clinical routines were mainly focused on a better case conceptualization and improved diagnostic understanding of the patient population. It appears that the improvement of diagnostics facilitated more appropriate allocation of patients to effective treatments and/or treatment planning. The design of our study does however not allow for a confirmation of this hypothesis. Future research should investigate the role of diagnostic accuracy and correct case conception on treatment outcome.

### Limitations

The results from study are limited by several aspects. First, the study design does not allow causal inference, and therefore we cannot rule out that other factors have influenced or biased our findings. Studies involving rigorous control conditions and designs that allow causal inference are therefore warranted. Second, as hypothesized previously, it appears that an improvement in diagnostic assessments contributed to an improvement of treatment outcomes. However, the study design does not allow an evaluation of this hypothesis. Mechanistic analyses or fidelity checks of the newly implemented diagnostic routines were not feasible in the current study, as it was conducted in regular mental healthcare with limited possibilities to burden clinicians with additional data collection routines. Therefore, eloquently designed studies that test the importance of accurate diagnostic assessments for treatment outcomes are warranted. Thirdly, the current study focused on depression treatment due to the initially sub-optimal results. It would be important to test the comprehensive quality improvement method used in this study in other contexts and clinical populations to gain better generalizability. In addition, the symptom severity in the clinical sample was higher than in the benchmark sample, and the clinical sample received more treatment sessions than the benchmark sample. This can be understood in the light of the clinical context, as the participating clinics were second-line, specialized clinics and would thus receive a high proportion of severely ill patients with high symptomatic burden, often requiring longer treatments. However, this imbalance between the clinical and the benchmark sample limits the direct statistical comparability in our analyses. Lastly, the primary outcome in our study was the PHQ-9, which was available with sufficient coverage as it had been implemented as a routinely collected PROM at the participating clinics. The availability of additional measures, e.g. functional impairment, satisfaction measures, adherence to treatment protocols, information on treatment types and combinations, as well as clinician-rated measures, would have been beneficial and had enabled us to conduct further analyses on relevant layers of clinical processes and outcomes.

## Conclusions

Results from this study indicate that the combination of MBC, routinely collected PROMs, benchmarking and CQI could improve results for depression treatments in specialized mental healthcare. As causal mechanisms remain unclear, these findings warrant replication in well-controlled study designs.

## Supplementary Information

Below is the link to the electronic supplementary material.

Supplementary Material 1

## Data Availability

Data is not available due to Swedish and EU law applicable to sensitive information.
