# Supplementary material for: Enhancing Clinical Depression Treatment Outcomes: A Comprehensive Approach Using Measurement-Based Care, Research Benchmarks, and Systematic Quality Improvements
Source: Adm Policy Ment Health. 2026 Mar 31;53(4):329–37. doi: 10.1007/s10488-026-01499-6 (PMC13372958; doi:10.1007/s10488-026-01499-6)
Supplement: Supplementary file 1 — Supplementary Material 1 [file 10488_2026_1499_MOESM1_ESM.docx]

# **Supplemental materials**

**Table S1.** Within-group (pre-post) effect sizes (Hedges’s *g*) for clinic depression participants divided by year and compared to overall meta-analytic benchmark sample.

| **Clinical results** | | | | **Meta-analytic benchmark** | | |
| --- | --- | --- | --- | --- | --- | --- |
| Year | n | ES | 95% CI | n | ES | 95% CI |
| 2019 | 110 | 0.94* | 0.72-1.16 | 3525 | 1.51 | 1.36-1.65 |
| 2020 | 62 | 0.87* | 0.58-1.16 |  |  |  |
| 2021 | 84 | 1.01* | 0.76-1.26 |  |  |  |
| 2022 | 97 | 1.25 | 0.98-1.51 |  |  |  |
| 2023 | 62 | 1.27 | 0.94-1.61 |  |  |  |

*Note*: n = number of participants, ES = effect size (Hedges’s *g*). CI = confidence interval. * = lower than meta-analytic benchmark.
